# Synthesis and Properties of Poly[*p*-(2,5-dihydroxy)-phenylenebenzobisoxazole] Fiber

**DOI:** 10.3390/ijms9112159

**Published:** 2008-11-05

**Authors:** Hong Lin, Yu-Dong Huang, Feng Wang

**Affiliations:** Department of Applied Chemistry, Faculty of Science, Harbin Institute of Technology, Harbin 150001, P.R. China. E-Mail: lin-hong2001@163.com

**Keywords:** Poly[p-(2,5-dihydroxy)-phenylenebenzobisoxazole], Polycondensation, Compressive strength, Hydrogen bonding

## Abstract

The novel polymer poly[*p*-(2,5-dihydroxy)-phenylenebenzobisoxazole] (PBOH) fiber was synthesized in the presence of 2,5-dihydroxyterephthalicacid (DHTA) and 4,6-diamino-1,3-benzenediol in poly(phosphoric acid) (PPA) using typical polycondensation conditions. The crystalline solutions of liquid PBOH in PPA were spun into fibers using dry-jet wet spinning. Furthermore, the thermostability and mechanical properties of PBOH were compared with poly(*p*-phenylene-2,6-benzoxazole) (PBO) in order to investigate the relationship between the chain structure and properties. The results indicated that the thermal degradation temperature of PBOH was above 750K and the tensile strength of the PBOH fiber was 3.1GPa, which were much lower than those of PBO fiber. The compressive strength of PBOH fiber was 331 M Pa, which was slightly higher than that of PBO fiber. In addition, molecular simulation was employed to explain why the compressive strength of PBOH fiber did not increase significantly compared to PBO fiber.

## 1. Introduction

Rigid-rod polymers, like poly(*p*-phenylene-2,6-benzoxazole) (PBO) fiber, have excellent thermal stability, solvent resistance, remarkable tensile strength and modulus [1–3]. However, their performance under compression has been disappointing. Much work has been done using various methods to conquer this problem. Most workers have focused on approaches that can enhance lateral interaction of polymers to increase the compressive strength of the fibers [4]. The methods included cross-linking by coupling of free radicals, imbedding sol-gel glass (or as thermoset matrix), synthesis of two dimensional PBO and coating with a stiff ceramic material [4–7]. Although the problem has apparently been solved with the crosslinking and intercalation approach, only modest changes in fiber compressive strength have resulted, at the expense of tensile strength according to some reports, so as far as producing PBO fibers to be used as reinforcing materials in structural composites is concerned, improvement of fiber compressive strength remains a challenge.

**Figure 1.**
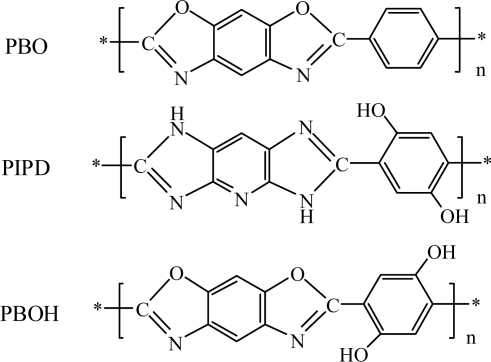
Structural formulae of PBO, PIPD and PBOH.

Recently, some literature has reported the novel polymer fiber poly[2,6-diimidazo (4,5-β–4',5'-ɛ)pyridinylene-1,4-(2,5-dihydroxy)phenylene] (PIPD or M5) which possesses somewhat higher compressive strength due to a unidirectional hydrogen bonding network (the structural formula is shown in Figure 1). However, the unidirectional action force of PBO or PBT is only due to weak Van der Wasls interactions and therefore, with the introdution of polar groups, intermolecular hydrogen bonding is formed, which can increase the intermolecular force between essentially rigid molecular chains so as to improve the compression behavior of composites [4, 8].

In this study, a novel poly[*p*-(2,5-dihydroxy)-phenylenebenzobisoxazole] (PBOH) polymer fiber has been synthesized for the first time, and was characterized by its intrinsic viscosity, TG-DTG, XRD and Raman. PBOH fiber spinning was done by a dryjet wet-spinning technique. The thermostability and mechanical properties of PBOH were compared with those of PBO in order to investigate the relationship between the chain structure and properties and molecular simulation was also employed to discuss the action force between the PBOH molecules.

## 2. Experimental

### 2.1. Materials

The PBO fiber was prepared according to the method reported in the literature [11]. The characteristics of the obtained PBO fiber were as follows: the intrinsic viscosity is 21.6 dL/g, the tensile strength is up to 4.5 GPa, and the tensile modulus is up to 260 GPa. 4,6-Diamino-1,3-benzenediol was prepared in our laboratory. Dimethyl-1,4-cyclohexanedione-2,5-dicarboxylate was purchased from WEIBO Chemical Co. and was recrystallized from toluene. Methanesulfonic acid (MSA) was obtained from Changzhou-PRILRIKE Chemical Co., and was distilled under reduced pressure.

### 2.2. Measurements

^1^H-NMR and ^13^C-NMR spectra of the synthesized monomer were recorded on a Bruker Fourier Transform AVANCE 400 spectrometer. The chemical shifts are reported in parts per million (ppm) using tetramethylsilane as an internal reference. Intrinsic viscosities of the synthesized polymer were measured in methanesulfonic acid (MSA) at 30 °C using a modified Ubbelohde capillary viscometer. Fourier transform infrared (FTIR) spectra were taken at room temperature using a Nicolet Nexus 670 FTIR spectrometer. Thermogravimetric analysis (NETZSCH STA 449C) was used for the analysis of weight loss of the PBOH. The sample (about 5 mg) was placed in Al_2_O_3_ crucibles, and the analysis was performed at heating rates of 10.0 K·min^−1^ from 293 K to 1473 K. Raman spectra for the PBOH fibers were obtained using a Renishaw RM2000 Raman system, as described earlier. The wide angle X-ray diffraction patterns were measured on a Rigaku D/MAX-2500V-PC diffractometer using Cu Ka radiation (40 kV, 100 mA, λ = 0.154 nm), filtered by Cr. The experiments were performed in a range of 2θ = 1.0–3.5° with a scan rate of 4°C/min. Fiber compressive strength was determined by the tensile recoil test [12]. For tensile testing, fibers were mounted on cardboard tabs. Testing was performed on an universal tensile tester (model WD-1) using 2.00 cm gage length at a strain rate of 2%/min. The fiber diameter was measured with a optical microscope (equipped with CCD, CAMERAL).

### 2.3. Synthesis

#### 2.3.1. Synthesis of 2,5-Dihydroxyterephthalic acid (DHTA) (Scheme 1)

In a 500 mL flask with stirrer, thermometer, reflux head and dropping funnel, dimethyl 1,4-cyclohexanedione-2,5-dicarboxylate (22.8 g, 100 mmol), was dispersed in acetic acid (220 mL). of Iodine (0.2 g) and potassium iodide (0.2 g) were added quickly to the dispersion when it was heated to 80 °C. Then 30% H_2_O_2_ (10 mL) was added when the temperature was reached 95–100 °C. After the addition of H_2_O_2_, warm and cold water (40 mL of each) were stirred into the reaction mixture. After cooling to room temperature, the slurry was filtered and washed twice with water, to give the yellow 2,5-dihydroxy-1,4-benzenedicarboxylic acid dimethyl ester product.

In a 500 mL flask with stirrer, dropping funnel, thermometer, reflux head and nitrogen inlet, NaOH (10 g, 250 mmol) was stirred into warm water (50 mL). An amount of dry 2,5-dihydroxy-1,4-benzenedicarboxylic acid dimethyl ester (22.6 g, 100 mmol) was slurried in water (80 mL), and the slurry was added to the NaOH solution. Under nitrogen, 36% HCl (20 mL) was added when the mixture reached 95 °C. After cooling to room temperature, the DHTA was filtered off, washed with water three times and dried at 80 °C for 20 h under vacuum. The product was recrystallized from CH_3_CH_2_OH/H_2_O to give a yellow powder (13.96 g, 71.1% yield). The product’s characteristics were as follows. FTIR (KBr, cm^−1^): wide band at 3,200–2,500 peaking at about 3,100, 1,650, 1,496, 1,429, 1,359, 1,292, 1,181, 899, 848, 698, 527; NMR (DMSO-*d_6_*): 1H δ 7.27; ^13^C δ 116.95, 118.97, 151.59, 168.94.

#### 2.3.2. Polymerization

In the synthesis of PBOH (shown in Scheme 2), 4,6-diamino-1,3-benzenediol (DADHB, 10.65 g, 5 mmol) and 2,5-dihydroxyterephthalic acid (DHTA, 9.90 g, 5 mmol) were added to degassed polyphosphoric acid (PPA) solution (83.5%, 61.86 g) in a glass reaction vessel which was protected under nitrogen.

After dehydrochlorination at 100–110 °C under vacuum, the reaction vessel was cooled down to about 90 °C. Fresh P_2_O_5_ (18.23 g) was added, and the reaction mixture was stirred for 60 min to ensure full mixing, then the reaction temperature was raised as follows: firstly, temperature was raised to 130 °C and held for 5–6 h, then to 150 °C for 6–7 h, in the end, to 190 °C for 3–4 h.

Under proper fiber spinning conditions, PBOH fiber was spun by dry-jetwet-spinning from the highly viscous PBO/PPA dope (brown in color) and purified by extraction of the PPA with water for 72 hours. The polymer fiber was desiccated at 60 °C in a vacuum oven. PBO was synthesized according to the report using a similar method [11].

## 3. Results and Discussion

### 3.1. Characteristics of the synthesized polymer

The polymerization reaction was carried out in a kettle reactor. Compared with the polymerization reaction of PBO, the solubility of DHTA is higher in PPA than that of TAP. Before the addition of P_2_O_5_, the DHTA is basically solubilized in PPA. Therefore, the reaction rate is little faster. In the polymerization process, it can be seen that the polymerization solution’s color is henna. The phenomenon of the polymer liquid crystallinity was appeared when the temperature is up to 170 °C.

Viscosimetry in MSA is used to determine the *M*_w_ of PBOH. An approximate *M*_w_ value is calculated using the relationship between intrinsic viscosity *η* and molecular weight for PBO [13, 14]. The *M*_w_ value and the polymerization reaction time are compared to the values of PBO (as shown in Table 1). These results clearly show that with the reaction rate increase, the molecular weight obtained become lower than PBO.

### 3.2. Thermal decomposition behaviour of PBOH fiber

**Figure 2.**
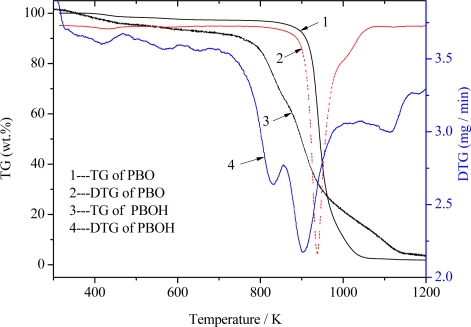
TG-DTG curve of PBOH fiber and TG curve of PBO.

**Figure 4.**
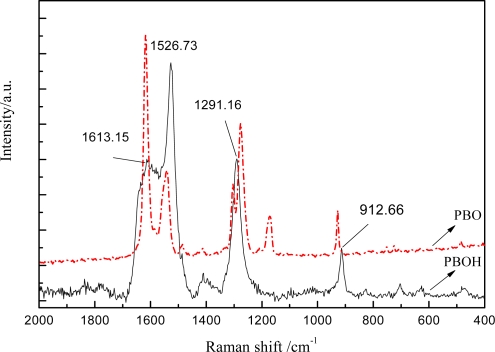
Raman spectroscopy of PBO and PBOH fiber.

Figure 2 shows the curve of TG-DTG of PBOH fiber at the heating rate of 10 K·min^−1^ under an air atmosphere. In order to compare the results with PBO fiber, Figure 4 also shows the TG curve of the latter. It can be seen from Figure 2 that the TG curves of PBOH fiber decreased slightly in the initial stages, which is due to the loss of water around 400 K. Under the air exposure conditions, the thermal degradation occurs above 750 K and finally forms about 3 wt% residue at 1,200 K, with significant changes happening between 800 and 1,000 K. On the other hand, for PBO fiber, the thermal degradation happened at about 850 K, and the significant changes happened between 800 and 1,100 K. It can be found that PBO fiber has better heat resistance in the atmosphere of air, which may be attributed to the highly conjugated system and great stiffness of the PBO molecule.

As shown the DTG curve of PBOH, the fastest decomposition speed happened at 904 K. And there are three peak values in the DTG curve of PBOH, which shows that the PBOH decomposition is a multistep process. The mechanism may be that the existence of hydroxyls decreases the thermal stability, which leads to the easy generation of thermal oxidation decomposition.

The thermal stability of PBOH is 100 K lower than PBO. This is because the presence of hydroxyls plays a critical role in the beginning of degradation in polymers [15]. These analyses indicate that hydroxyls have a strong influence on the thermal degradation of the PBOH fibers and that the decomposition reactions are different between PBOH fiber and PBO fiber.

### 3.3. XRD and Raman analysis of PBOH fibers

The curve of XRD for fresh PBOH fiber (without the step of heat drawing) and Zylon-AS PBO fiber is shown in Figure 3. It can be seen that the PBOH fiber’s diffraction angle 2θ is 15.96 and 26.02 respectively, which is almost similar to the diffraction angle of PBO fiber.

Formation of PBO and PBOH fiber involves a transition from a nematic phase in solution to a polymer solid state, which occurs during coagulation due to deprotonation of the polymer by the coagulant. Crystal-solvate phase [16] is a co-crystallization product of the polymer and its solvent (water in this case) as coagulant, therefore, the crystal solvate is an intermediate phase between the nematic solution and the solid crystal phase. The PBOH fiber with hydroxyl pendant groups, very easily formed a solvate with water. Without heat-treatment the fiber axial distance between the boundary of one crystal and the boundary of the next crystal is a little longer, crystal degree is lower, and average crystal size is smaller, which are generally attributed to the presence of voids or crystals elongated along the fiber axis. Due to the removal of solvent during coagulation a drastic volume reduction is a significant cause for the presence of voids. Fresh fibers are usually treated through a postspinning heat drawing in order to enhance fiber tensile mechanical properties.

Figure 4 shows the Raman spectra in the 800–2,000 cm^−1^ range for fresh PBOH and PBO fibers. The Raman spectrum of Zylon AS (not shown) is equivalent. The spectrum of the fresh PBOH fiber exhibits several well-defined Raman-active bands, four of them of high intensity (1,613 cm^−1^, 1,526 cm^−1^, 1,291 cm^−1^ and 912 cm^−1^).

According to the chemical composition and the structure of this polymer, the following band assignments can be made: 1,613 cm^−1^, ring stretching vibration of the benzene ring in the heterocycle (benzobisoxazole) as well as the phenylene ring coupled with the stretching vibration of the C-C bond connecting benzobisoxazole and phenylene; 1,526 cm^−1^, ring stretching vibration of the phenylene group coupled with ring deformation vibration of benzene in the benzobisoxazole group; 1,391 cm^−1^, phenylene ring deformation, C-C stretching of the carbons linking the heterocycle and the phenyl ring; and 930 cm^−1^, C-H out of plane bending vibrations in the phenylene ring.

The spectrum of the fresh PBO fiber exhibits six well-defined Raman-active bands (1,620 cm^−1^, 1,545 cm^−1^, 1,307 cm^−1^, 1,290 cm^−1^, 1,174 cm^−1^ and 912 cm^−1^). Compared with the PBO fiber, the 1,174 cm^−1^ peak disappeared, the broad component 1,307 cm^−1^ and 1,290 cm^−1^ peaks have also vanished and the 1,291 cm^−1^ peak appered; other peaks are located at the same regions, and differ only in their relative intensity.

### 3.4. Mechanical properties of PBOH fibers

The surface of the obtained PBOH fibers is smooth and turned out to be red metallic luster. The mechanical properties of PBOH and PBO fibers are shown in Table 2.

The compressive strength of PBO fiber is lower due to the fibril which consists of the rigid molecular chain of PBO shifts and slips very easily by compression. Therefore, the molecular chain cannot maintain the compression force in the fiber-axis direction. Increasing the interaction of interchain can reduce the slips during the compression. Thus hydroxyl groups introduced in can provide hydrogen bonding among polymer chains, which can provide lateral reinforcement across the fiber to increase the compressive strength of the fiber.

However, as shown in Table 2 the tensile strength of the PBOH fibers is lower than that of PBO fibers, while the compressive strength of the PBOH fibers is higher than that of PBO fiber. The increase of the compressive strength, is not as significant as expected, The compressive strength achieved is still much smaller than that of Kevlar (0.35–0.45 GPa) and M5 (1.6 GPa)[4].

Ref. [17] reports a PBT fiber with hydrogen bonding. Compressive strength values of this PBT fiber are very scattered within each group of fiber. The authors attribute the failure of the hydroxyl pendant groups to improve fiber compressive strength to hydrogen bonding occurring in an intrachain fashion[4]. Thus, a pseudo-ladder structure rather than the desired interchain hydrogen-bonded structure is proposed.

### 3.5. Molecular simulation of PBOH

**Figure 5.**
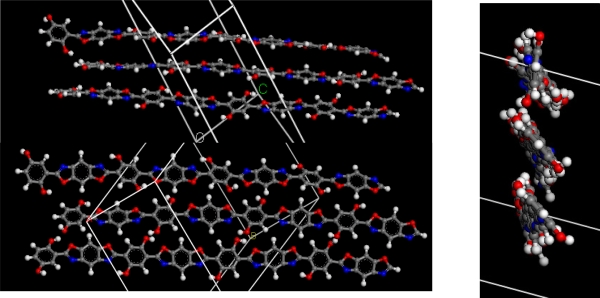
Result of molecular structure (several different angles).

The Materials Studio software was employed to do a molecular simulation for PBOH. Figure 5 is the molecular structure of PBOH built by the Periodic Boundary Model, and the degree of polymerization is four. The hydrogen bonding among polymer chains which is produced by the hydroxyl groups is also shown in Figure 5. However, the hydrogen bonds only exist on the side of N atom in the benzobisoxazole group except on the side of O atom. In addition, the stereospecific blockade is produced by the hydrogen bonds between the intermolecular, so the chains are not easy to approach which resulted in the easiness of inter-chains shifts and slips by tension or compression. These reasons possibly results in the undesirable effect. Thus, the further improvement of the molecular structure and the intermolecular force is necessary. The work will be studied in-depth in our lab. It is necessary to improve the molecular structure for preventing the sliding of the intermolecular.

## 4. Conclusions

In order to improve the PBO fiber compressive strengths, the hydroxyl pendent groups were introduced into the PBO chain.Thereby we prepared the PBOH fiber.

Through thermal analysis, it is found that the thermal degradation of PBOH occurs above 750 K. And the thermal stability of PBOH fiber is 100 K lower than that of PBO. Through mechanical analysis, it is found that the tensile strength of the PBOH fiber is 3.1 GPa, and the value of compressive strength is little higher than that of PBO fiber. Throuh the molecular simulation, it can be found that the hydroxyl groups which was introduced into the PBO chain were not formed the optimal hydrogen bonding. So it is necessary to improve the molecular structure to prevent the sliding of the intermolecular.

## Figures and Tables

**Figure 3.**
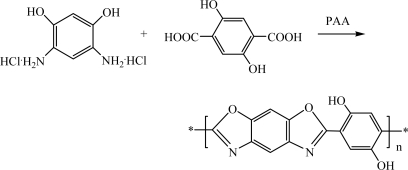
Synthesis of PBOH fiber.

**Figure 3.**
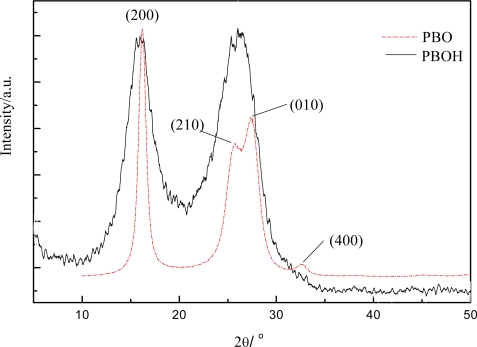
XRD curves of PBO and PBOH fiber.

**Scheme 1.**
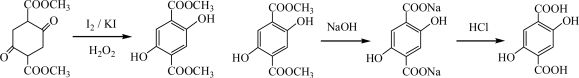
Synthesis of DHTA.

**Table 1.** The comparison of viscosities and molecular weight between the PBOH and the PBO.

	PBOH	PBO
Reaction time/h	15~18	24~26
[*η*]/dL·g^−1^	18.1	21.6
*M̄_w_*	78000	84600

**Table 2.** Mechanical properties of PBOH and PBO fibers.

	PBOH	PBO	Zylon-AS
Tensile strength / GPa	3.1 ± 0.2	4.2 ± 0.2	5.8 ± 0.1
tensile modulus / GPa	155 ± 7	174 ± 5	190 ± 4
Fibre Diameter */*μ *m*	18–20	18–20	11–13
compressive strength / mPa	331 ± 10	277 ± 9	297 ± 4
